# Elevated glucose and oligomeric β-amyloid disrupt synapses via a common pathway of aberrant protein *S*-nitrosylation

**DOI:** 10.1038/ncomms10242

**Published:** 2016-01-08

**Authors:** Mohd Waseem Akhtar, Sara Sanz-Blasco, Nima Dolatabadi, James Parker, Kevin Chon, Michelle S. Lee, Walid Soussou, Scott R. McKercher, Rajesh Ambasudhan, Tomohiro Nakamura, Stuart A. Lipton

**Affiliations:** 1 Center for Neuroscience and Aging Research, Sanford Burnham Prebys Medical Discovery Institute, 10901 North Torrey Pines Avenue, La Jolla, California 92037, USA; 2 Neurodegenerative Disease Center, Scintillon Institute, 6868 Nancy Ridge Drive, San Diego, California 92121, USA; 3 Quantum Applied Science and Research, 5754 Pacific Center Blvd. Suite 203b, San Diego, California 92121, USA; 4 Department of Neurosciences, University of California, San Diego, School of Medicine, 9500 Gilman Drive, La Jolla, California 92093, USA

## Abstract

Metabolic syndrome (MetS) and Type 2 diabetes mellitus (T2DM) increase risk for Alzheimer's disease (AD). The molecular mechanism for this association remains poorly defined. Here we report in human and rodent tissues that elevated glucose, as found in MetS/T2DM, and oligomeric β-amyloid (Aβ) peptide, thought to be a key mediator of AD, coordinately increase neuronal Ca^2+^ and nitric oxide (NO) in an NMDA receptor-dependent manner. The increase in NO results in *S*-nitrosylation of insulin-degrading enzyme (IDE) and dynamin-related protein 1 (Drp1), thus inhibiting insulin and Aβ catabolism as well as hyperactivating mitochondrial fission machinery. Consequent elevation in Aβ levels and compromise in mitochondrial bioenergetics result in dysfunctional synaptic plasticity and synapse loss in cortical and hippocampal neurons. The NMDA receptor antagonist memantine attenuates these effects. Our studies show that redox-mediated posttranslational modification of brain proteins link Aβ and hyperglycaemia to cognitive dysfunction in MetS/T2DM and AD.

## Abstract (web summary)

Alzheimer's disease is linked to metabolic syndrome and Type-2 diabetes, but the mechanism behind this association is unclear. Here, the authors show that elevated glucose and amyloid ß work together to increase nitrosative stress, leading to aberrant mitochondrial activity and synaptic dysfunction.

## Graphical abstract

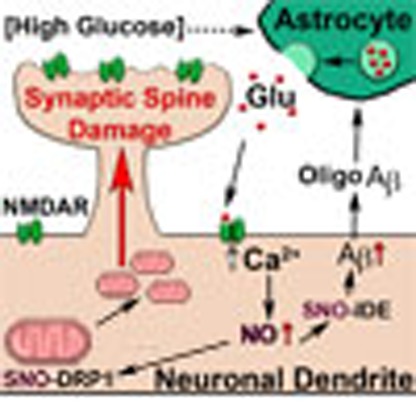

Obesity is a major predisposing factor for metabolic syndrome, and it has been estimated that as many as 30–40% of the population in Western countries is at risk. Epidemiological studies have shown a strong association between the risk of developing type 2 diabetes mellitus/metabolic syndrome (T2DM/MetS) and AD^1^,^2^. In addition, studies using rodent models of prediabetes, MetS, or T2DM manifest a number of molecular, synaptic and behavioural deficits in common with AD^3^,^4^. However, the mechanism for the linkage between these metabolic and cognitive diseases remains poorly understood. The basis of the association may be complex and involve many factors, including hyperglycaemia, insulin resistance, inflammation and altered lipid mediators^5^,^6^.

Here we report a major mechanism for the crosstalk between these diseases involving nitrosative stress that coordinately disrupts protein function. The mechanistic basis of these findings is that both diseases are oxidizing, altering the redox state of cortical and hippocampal neurons in the brain. Heretofore, glucose was known to be metabolized by oxidative phosphorylation with generation of oxygen free radicals/reactive oxygen species (ROS)^7^,^8^. In the present study, we find that increased levels of glucose also result in a marked rise in neuronal reactive nitrogen species (RNS), similar to our recent findings with oligomeric β-amyloid peptide (Aβ)^9^. In addition, we show that high glucose plus Aβ, as found in the brains of patients who manifest T2DM/MetS with AD, act coordinately to further increase RNS species to a greater degree than either one alone. The resulting nitrosative stress causes aberrant *S*-nitrosylation reactions on specific proteins^10^,^11^, which lead to additional increases in Aβ and glucose levels, compromise in mitochondrial function, and cause synaptic damage. Importantly, we also found that sucrose feeding-induced hyperglycaemia, as seen in T2DM/MetS, compromises hippocampal synaptic plasticity in a mouse model of AD. These results provide mechanistic insight into deficits in hippocampal-dependent learning and memory observed in such models of T2DM/MetS in the setting of AD^12^.

## Results

### High glucose and Aβ oligomers induce nitrosative stress

**Figure 1:**
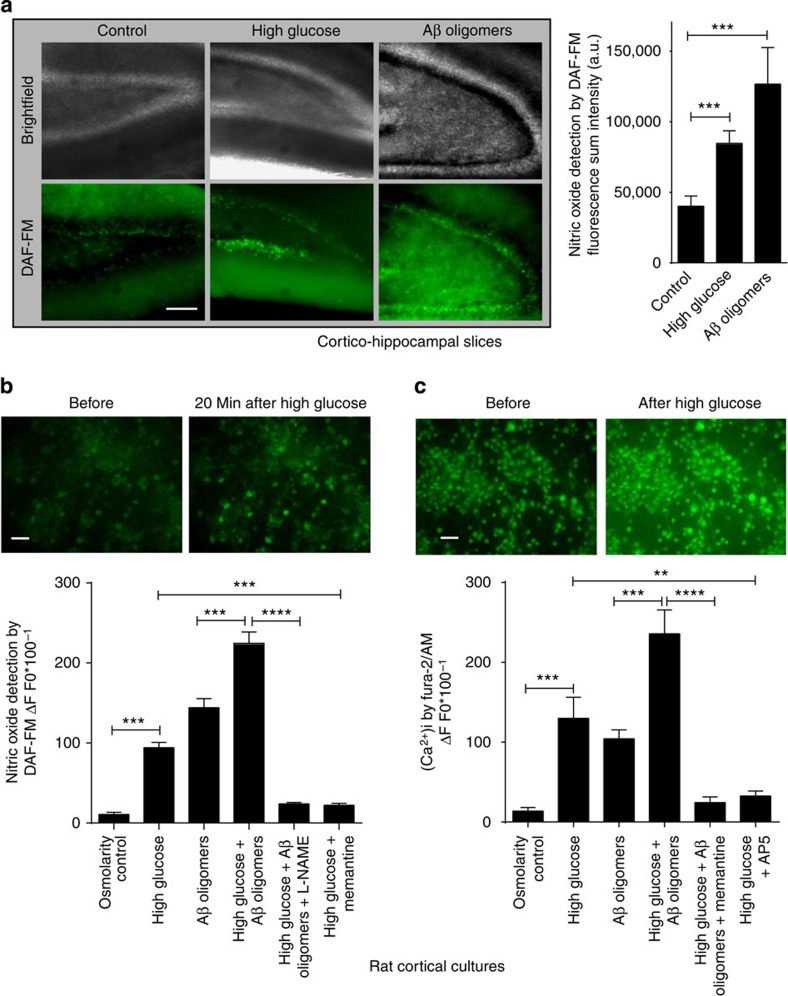
High glucose levels and oligomeric Aβ increase nitric oxide (NO) and neuronal calcium [Ca^2+^]_i_ in acute cortico-hippocampal slices and cortical neuronal cultures. (**a**) High glucose (25 mM) or oligomeric Aβ (250 nM) increase NO levels in acute cortico-hippocampal slices, monitored with DAF-FM imaging. Values are mean+s.e.m., *n*≥3; ***P<0.001 by ANOVA with Dunnett's *post hoc* test (Scale bar, 50 μm). (**b**) Quantitative DAF-FM imaging of cortical neurons showing increased NO after exposure to high glucose (20 mM above ambient levels) or Aβ oligomers (250 nM). Memantine (10 μM) inhibited the increase in NO in response to high glucose. Combined exposure to high glucose and Aβ manifested additive effects on NO that were inhibited by treatment with L-NAME (1 mM). Mannitol (20 mM) was used as a control for possible effects of osmolarity. For this and subsequent panels, fluorescence intensity change was calculated as Δ*F*/*F*0, plotted as a fraction of 100. Values are mean+s.e.m., *n*≥40 neurons for each condition; **P<0.01, ***P<0.001, ****P<0.0001 by ANOVA with Dunnett's *post hoc* test (Scale bar, 20 μm). (**c**) High glucose enhances Aβ-induced increases in [Ca^2+^]_i_ in cultured rat primary cortical neurons, monitored with fura-2/AM (*n*=∼15 cells for each condition); memantine (10 μM) or AP5 (100 μM) blocked the increase. Values are mean+s.e.m., *n*≥40 neurons for each condition; **P<0.01, ***P<0.001, ****P<0.0001 by ANOVA with Dunnett's *post hoc* test (Scale bar, 20 μm).

To assess nitrosative stress under conditions mimicking the hyperglycaemia of T2DM/MetS and the increased Aβ_1−42_ production of AD^13^, we initially exposed cortico-hippocampal brain slices to elevated glucose or oligomeric Aβ_1−42_ and monitored neuronal nitric oxide (NO) levels using the probe 4-amino-5-methyamino-2′, 7′-difluorofluorescein (DAF-FM) diacetate. We found a robust increase in NO in cortico-hippocampal neurons incubated in artificial cerebrospinal fluid (ACSF) containing either high glucose (25 mM) or oligomeric recombinant Aβ_1−42_ (250 nM) compared with normal ACSF alone (with 10 mM glucose; Fig. 1a). A similar increase in NO was observed in cortical neurons cultured for 14–21 days, a stage at which these cells manifest a variety of glutamate receptors and relatively mature synapses (Fig. 1b)^14^,^15^. As a control for potential osmotic effects of added glucose, equimolar mannitol did not increase NO. In addition, combining high glucose with oligomeric Aβ_1−42_ coordinately increased NO to a greater degree than either alone. This increase was inhibited by incubation in the NO synthase (NOS) inhibitor, L-N^G^-nitroarginine methyl ester (L-NAME, 1 mM; Fig. 1b). Collectively, these results suggest that high glucose and Aβ exposure cause an NOS-mediated increase in NO in both cortico-hippocampal brain slices and cortical cultures.

### High glucose and Aβ oligomers increase neuronal Ca^2+^ and NO

Next, we examined the upstream cellular events that mediate the increase in NO in cortical neurons exposed to high glucose and Aβ oligomers. Calcium influx via *N*-methyl-D-aspartate receptor (NMDARs) is known to activate neuronal (n)NOS^16^. Therefore, we studied whether high glucose or oligomeric Aβ could increase neuronal intracellular calcium ions [Ca^2+^]_i_ in an NMDAR-dependent manner. Indeed, we observed an increase in [Ca^2+^]_i_ in cortical neurons exposed to high glucose and oligomeric Aβ that was largely abated by the specific NMDAR antagonists (2*R*)-amino-5-phosphonovaleric acid (AP5) or memantine (Fig. 1c)^9^. Note also that combined exposure to high glucose and Aβ manifested an additive increase in [Ca^2+^]_i_. Furthermore, exposure to memantine blocked the increase in NO engendered by high glucose (Fig. 1b), similar to its inhibitory effect on oligomeric Aβ-induced increases in NO, as we recently reported^9^. These data are consistent with the notion that NMDAR-mediated increases in [Ca^2+^]_i_ result in nNOS activation and consequent generation of NO in response to high glucose or oligomeric Aβ.

In further support of the above premise, we and others have previously found that oligomeric Aβ enhances glutamate release from both neurons and astrocytes^9^,^17^. Moreover, high glucose is also known to increase glutamate release in cerebral synaptosomes prepared from diabetic mice^18^. In addition, glutamate levels are higher in human brains with MetS than in control brains, as monitored by magnetic resonance spectroscopy^19^. Taken together, these findings suggest that high glucose- or Aβ-mediated increases in extracellular glutamate result in NMDAR activation, accounting at least in part for the observed increase in neuronal [Ca^2+^]_i_, nNOS stimulation, and consequent nitrosative stress.

### High glucose or Aβ oligomers *S*-nitrosylate and inhibit IDE

**Figure 2:**
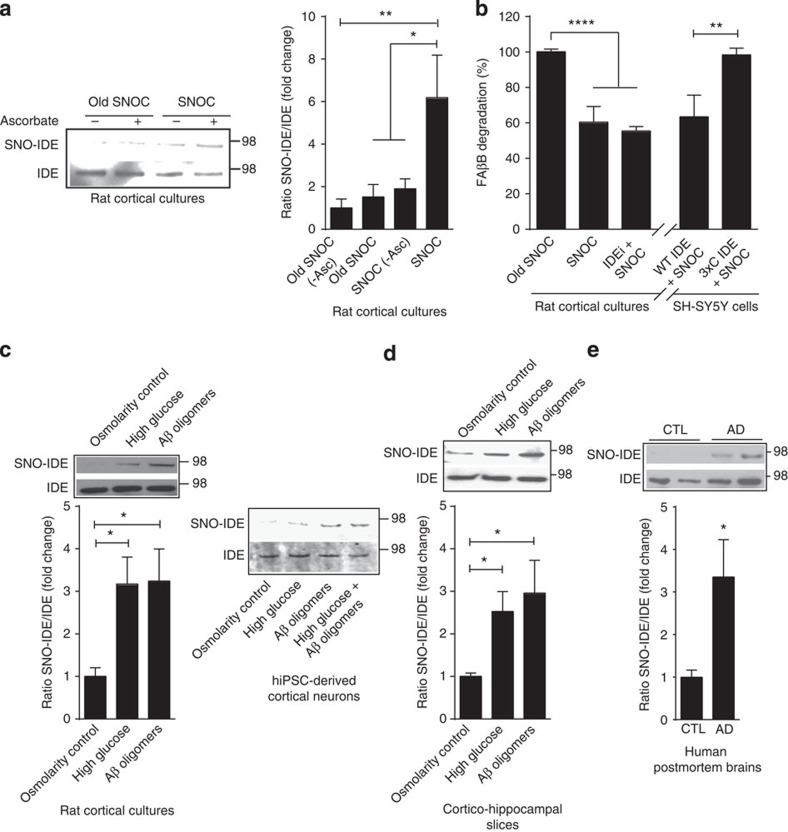
*S*-Nitrosylation and inhibition of neural IDE. (**a**) SNO-IDE in cortical cultures. Biotin switch assay after 30-min exposure to 200 μM SNOC. Bottom panel, loading control. Densitometry shown on right. As a control, sodium ascorbate (20 mM) was omitted. Values are mean+s.e.m., *n*=3; *P<0.05, **P<0.01 by ANOVA with Fisher's Protected Least Significant Difference (PLSD) *post hoc* test. (**b**) *S*-Nitrosylation of IDE inhibits its activity. Left: less FAβB peptide degradation in cortical lysates following cell exposure to SNOC. Addition of the IDE-specific inhibitor, IDEi, produced similar results. Right: non-nitrosylatable IDE rescues IDE activity in SH-SY5Y cells after SNOC exposure. Overexpressed WT-IDE, but not non-nitrosylatable (triple-cysteine mutant) IDE, significantly decreased FAβB after SNOC exposure. Values are mean+s.e.m., *n*≥3; **P<0.01, ****P<0.0001 by ANOVA with Tukey's *post hoc* test. (**c**) High glucose and oligomeric Aβ induce SNO-IDE formation in cortical cultures and in hiPSC-derived neurons by biotin switch assay. Cortical cultures (15 days *in vitro* (DIV)) or hiPSC-derived neurons (100 days post differentiation) were exposed to high glucose (20 mM) or oligomeric Aβ (250 nM) for 2 h. Equimolar mannitol served as an osmolarity control for high glucose. Values are mean+s.e.m., *n*=3, *P<0.05 by ANOVA with Fisher's PLSD *post hoc* test. (**d**) Acute rat cortico-hippocampal slices were exposed to high glucose, equimolar mannitol osmolarity control or oligomeric Aβ for 2 h. Biotin switch detected SNO-IDE. Values are mean+s.e.m., *n*=3, *P<0.05 by ANOVA with Fisher's PLSD *post hoc* test. (**e**) Detection of *S*-nitrosylated (SNO-) IDE in human post-mortem brain lysates by biotin switch. Values are mean+s.e.m., *n*≥6, *P<0.05 by Student's *t*-test.

Our group has previously shown that nitrosative stress causes aberrant *S*-nitrosylation of several important cellular proteins, thereby compromising their function^10^,^20^. Hence, we reasoned that high-glucose- and Aβ-induced nitrosative stress might cause aberrant protein *S*-nitrosylation in the context of T2DM/MetS and AD. One critical protein thought to be important in both T2DM/MetS and AD is insulin-degrading enzyme (IDE), a Zn-metalloendopeptidase that hydrolyses specific biological peptides including Aβ and insulin^21^,^22^. Decreased IDE function results in increased Aβ and insulin levels, as observed in T2DM/MetS and AD^23^,^24^. Moreover, several prior reports have shown that recombinant IDE can be oxidized, alkylated or *S*-nitrosylated at identified cysteine residues, and that each of these modifications inhibits its enzymatic activity, at least *in vitro*^25^,^26^. To investigate whether IDE can be *S*-nitrosylated *in situ* in neurons, we initially exposed cortical cultures to the physiological NO donor *S*-nitrosocysteine (SNOC). Using the biotin switch assay^27^, we found that SNOC, but not old SNOC from which NO had been dissipated, increased *S*-nitrosylation of IDE, forming SNO-IDE (Fig. 2a). These results show that IDE can be a target for *S*-nitrosylation in neurons.

Since we have shown that high glucose and oligomeric Aβ can increase NO, we next asked whether the glucose or Aβ levels found in T2DM/MetS and AD could generate a sufficient degree of endogenous NO to *S*-nitrosylate and thus inhibit IDE activity. If so, then this would result in a pathological positive-feedback-loop to further augment Aβ and insulin levels. For these experiments, we exposed primary rat cortical cultures and acute rat cortico-hippocampal slices to high glucose and/or oligomeric Aβ. Importantly, we also used human-induced pluripotent stem cells (hiPSC)-derived cortical neurons to ensure that the observed effects occurred in human context as well. Biotin switch assay revealed that SNO-IDE increased significantly in the presence of high glucose or oligomerized Aβ in all three preparations (Fig. 2c,d). In fact, the combination of high glucose and oligomeric Aβ produced the greatest increase in SNO-IDE in hiPSC-derived cortical cultures (Fig. 2c).

To determine whether NO could inhibit IDE activity to affect Aβ catabolism in neural cells, we employed a fluorescein-Aβ-biotin or 5-carboxyfluorescein-Aβ-biotin (FAβB) assay in which the intensity of fluorescence indicates Aβ degradation^28^. Using this assay, we observed a significant decrease in Aβ degradation in cortical neurons after exposure to SNOC. Incubation in an IDE inhibitor^29^ occluded further Aβ degradation by SNOC (Fig. 2b, left-hand panel), consistent with the notion that NO inhibited IDE activity. To further quantify the contribution of IDE to Aβ degradation using this assay and to assess the effect of *S*-nitrosylation, we used a non-nitrosylatable mutant of IDE in which the three active cysteine residues that undergo reaction with NO are converted to alanines^25^,^26^. When we overexpressed wild-type IDE or the non-nitrosylatable triple-cysteine mutant (3 × C) IDE in SH-SY5Y human neural cells, SNOC decreased Aβ degradation only in the wild-type (Fig. 2b, right-hand panel). Taken together with prior reports^25^, these results are consistent with the notion that *S*-nitrosylation of IDE inhibits enzymatic activity and thus Aβ degradation in a neuronal context.

### Aberrant SNO-IDE in human Alzheimer's disease brain

To extend these findings to the intact human brain, we examined post-mortem samples obtained shortly after death from patients manifesting sporadic AD versus controls (Supplementary Table 1). We observed significantly higher levels of SNO-IDE in AD brains than in age-matched controls (Fig. 2e; Supplementary Fig. 1). To determine whether this level of SNO-IDE in AD human brains is pathophysiologically important, we used a technique that we had previously described to calculate the ratio of SNO-IDE (determined by biotin switch assay) to total IDE (from immunoblots)^30^. This ratio was comparable to that encountered in our cell- and brain slice-based models (Fig. 2c,d). We subsequently showed in these model systems that this amount of SNO-IDE/total IDE, induced by the same concentrations of high glucose and/or oligomeric Aβ, was associated with synaptic damage (Fig. 4, below). Hence, collectively these findings are consistent with the notion that pathophysiologically relevant amounts of SNO-IDE are present in human AD brains. This result also suggests that SNO-IDE can potentially serve as a biomarker for AD.

### Synaptic loss associated with high glucose and Aβ oligomers

Analogous to our findings with SNO-IDE, we had previously reported in human AD brains the presence of pathophysiologically relevant levels of aberrantly *S*-nitrosylated dynamin-related protein 1 (Drp1), a GTPase responsible for mitochondrial fission^30^. Further, we showed that Aβ-induced *S*-nitrosylation of Drp1 in neurons caused hyperactivation of the fission machinery, resulting in excessive mitochondrial fragmentation with energy compromise and consequent synaptic damage^30^. Such perturbations in mitochondrial dynamics, representing aberrant fission and fusion events, have recently been implicated in a number of diseases with mitochondrial dysfunction, including AD and T2DM^31^,^32^.

**Figure 3:**
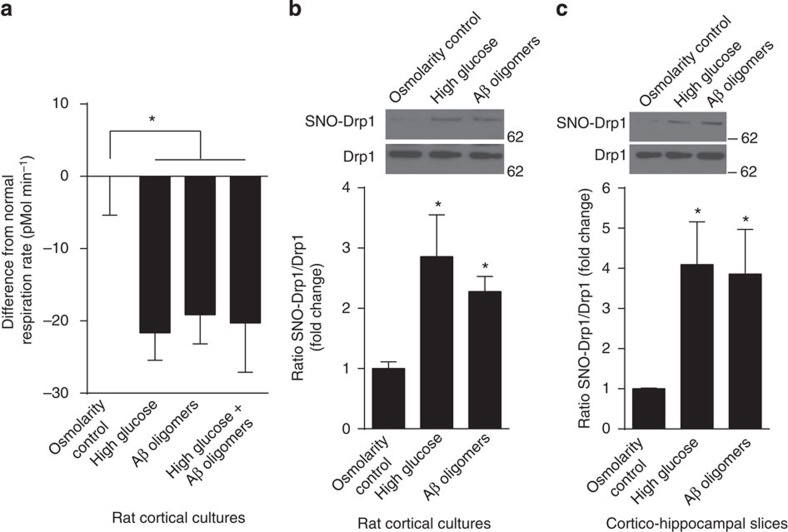
High glucose and oligomeric Aβ induce formation of SNO-Drp1 in cortical cultures and cortico-hippocampal slices and impair respiratory reserve. (**a**) Mitochondrial O_2_ consumption by cortical cultures was monitored using the Seahorse platform after exposure to high glucose (20 mM), equimolar mannitol as an osmolarity control or oligomeric Aβ (250 nM) for 72 h. Basal respiratory rate was measured in triplicate. Values are mean+s.e.m., *n*≥12, *P<0.05 by ANOVA with Tukey's *post hoc* test. (**b**) Cortical cultures (15 DIV) were exposed to high glucose (20 mM) or oligomeric Aβ (250 nM) for 2 h. Equimolar mannitol served as an osmolarity control for high glucose. Lysates were then analysed by biotin switch for SNO-Drp1. Values are mean+s.e.m., *n*≥3, *P<0.05 by ANOVA with Fisher's PLSD *post hoc* test. (**c**) Acute rat cortico-hippocampal slices were exposed to high glucose, equimolar mannitol osmolarity control or oligomeric Aβ for 2 h. Biotin switch detected SNO-Drp1. Values are mean+s.e.m., *n*=3, *P<0.05 by ANOVA with Fisher's PLSD *post hoc* test.

Here we asked if high glucose levels could compromise neuronal mitochondrial function in cortical cultures via excessive production of NO in a similar manner to that previously described for Aβ^30^,^33^. We monitored mitochondrial function by measuring O_2_ consumption using the Seahorse platform. We found that basal mitochondrial respiration rates were significantly decreased after exposure to high glucose, oligomeric Aβ or high glucose plus Aβ (Fig. 3a). Next, we determined whether this compromised mitochondrial function was associated with increased formation of neuronal SNO-Drp1. When we incubated cortical cultures or cortico-hippocampal slices in either high glucose or oligomeric Aβ_1−42_, we observed an increase in the ratio of SNO-Drp1/total Drp1 to levels comparable to those found in human AD brains (Fig. 3b,c)^30^.

**Figure 4:**
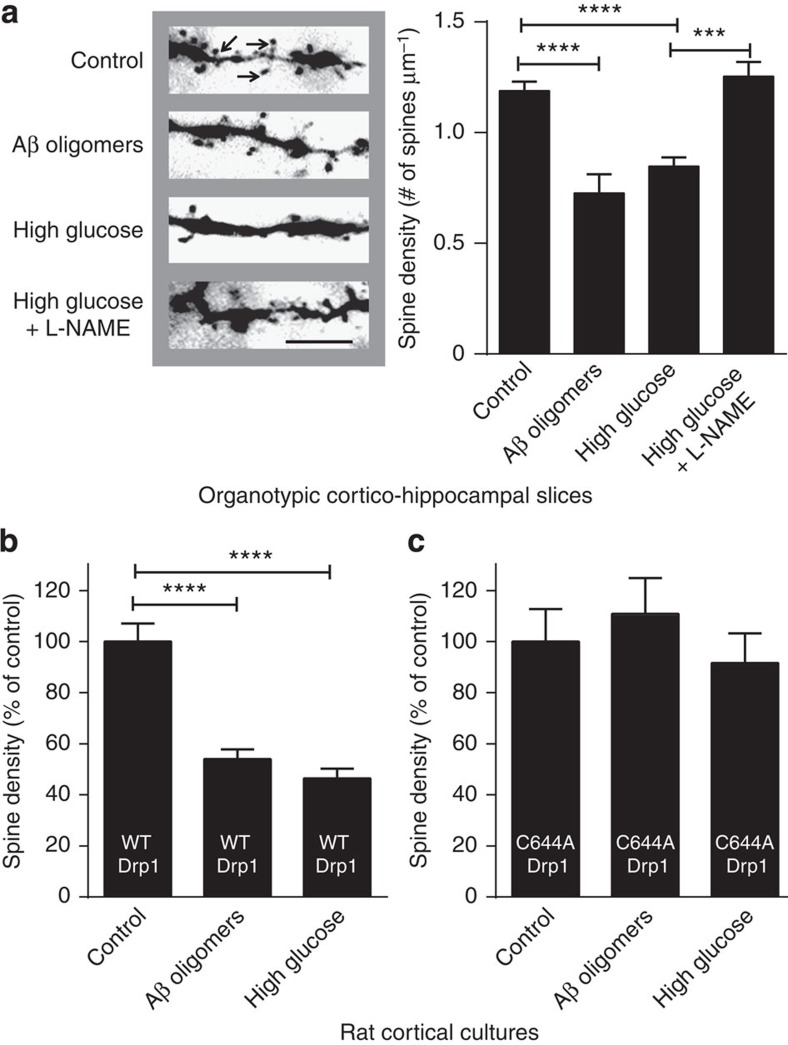
High glucose and oligomeric Aβ decrease dendritic spine density in a Drp1-dependent manner. (**a**) Dendritic spine density in organotypic cortico-hippocampal slices from YFP-transgenic mice after a 7-day exposure to high (25 mM) glucose or oligomeric Aβ (250 nM). Left: images of YFP-labelled dendritic spines (arrows) under epifluorescence microscopy (Scale bar, 10 μm). Right: quantification of spine density. The NOS inhibitor L-NAME (1 mM) prevented the decrease in spine density engendered by high glucose. Values are mean+s.e.m., *n*≥4 for each group, ***P<0.001, ****P<0.0001 by ANOVA with Tukey's *post hoc* test. (**b**) Overexpression of WT Drp1 did not substantially attenuate the spine loss due to high glucose of oligomeric Aβ in cortical cultures. Values are mean+s.e.m., *n*≥6, ****P<0.0001 by ANOVA with Tukey's *post hoc* test. (**c**) Overexpression of non-nitrosylatable Drp1 (C644A) mutant during exposure to high glucose or oligomeric Aβ resulted in spine number that was not statistically different from control. Values are mean+s.e.m., *n*≥10.

Synaptic loss is the major correlate of cognitive decline in human AD^34^,^35^. Since we had already shown that Aβ could induce synaptic loss via SNO-Drp1 formation^30^, we next investigated the effect of high glucose levels on synaptic damage. To quantify the effect of high glucose and compare it with that of oligomeric Aβ on synaptic spine density, we used Thy1-YFP transgenic mice in which a subset of hippocampal CA1 neurons expresses yellow fluorescent protein (YFP)^36^. We prepared organotypic cortico-hippocampal slices from these mice and exposed them to either oligomeric Aβ or high glucose in the presence or absence of 1 mM L-NAME. We found a significant, NOS-dependent decrease in spine density in slices exposed to high glucose (Fig. 4a) or Aβ oligomers^30^, suggesting that the decrease in spine number under these conditions is mediated at least in part by NO. Moreover, transfection of cortical neurons with mutant Drp1(C644A) lacking the nitrosylation site^30^, but not wild-type Drp1, abrogated the effect of high glucose or oligomeric Aβ on spine density (Fig. 4b,c). These findings are consistent with the notion that *S*-nitrosylation of Drp1 contributes to loss of synapses after exposure to either high glucose or oligomeric Aβ.

### Memantine rescues dendrites and spines in T2DM mice

The *in vitro* data described above suggest that exposure of rat cortical cultures or cortico-hippocampal slices to high glucose results in synaptic loss due to nitrosative stress triggered by Ca^2+^ influx via NMDARs. Previously, hippocampal synaptic spine density and cognitive function have been reported to be decreased *in vivo* in leptin receptor mutant (db/db) mice, representing a robust animal model of T2DM, compared to heterozygous (db/+) controls, which do not normally develop diabetes^37^,^38^. Therefore, we hypothesized that decreasing NMDAR activity would increase synaptic spine density in the db/db T2DM mice.

**Figure 5:**
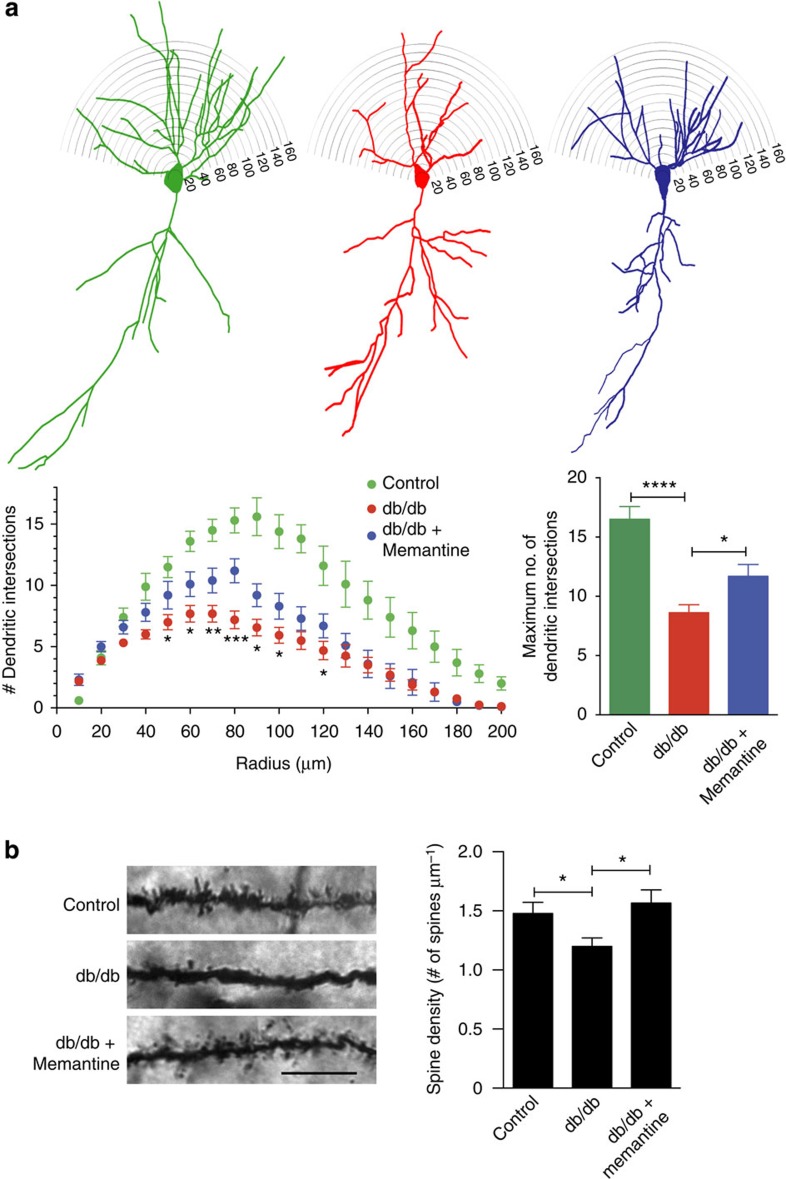
Chronic memantine treatment rescues dendritic arborization and synaptic density in a T2DM mouse model. (**a**) db/db mice show a significant decrease in dendritic branching in CA1 pyramidal neurons compared to heterozygous (db/+) controls. Chronic memantine treatment partially rescues the defect. Upper: representative delineated profiles of CA1 hippocampal pyramidal neurons with dendritic tree. Lower left: plot of dendritic intersections as a function of radius. Values are mean+s.e.m., *n*≥10 neurons from each of three mice, *P<0.05, **P<0.01, ***P<0.001 by ANOVA using repeated measures with Fisher's PLSD *post hoc* test. Lower right: quantification of total dendritic branching. Values are mean+s.e.m., *n*≥10 neurons from each of three mice, ****P<0.0001, *P<0.05 by ANOVA with Tukey's *post hoc* test. (**b**) db/db mice show a significant decrease in synaptic spine density in CA1 pyramidal neurons compared with controls. Chronic treatment with memantine completely reversed the loss of spines. Left: representative images of Golgi-stained dendrites from CA1 pyramidal neurons (Scale bar, 10 μm). Right: quantification of dendritic spine densities. Values are mean+s.e.m., *n*≥12 dendrites from three mice each, *P<0.05 by ANOVA with Fisher's PLSD *post hoc* test.

To test our hypothesis, we treated db/db mice with memantine (1 mg kg^−1^, twice daily) for 3 months. Initially, we examined dendritic arborization in this mouse model since the dendritic marker, microtubule-associated protein 2 (MAP2), has been shown to be decreased in the brains of db/db mice^38^. To study the effects of chronic memantine treatment on dendritic arborization in this model, we performed morphometric Sholl analysis on Golgi-impregnated CA1 hippocampal pyramidal neurons. We found a significant decrease in basal dendritic density (‘dendritic maxima'), as evidenced by a decrease in number of dendritic intersections in db/db mice compared with controls. This decrease in dendritic branching in db/db mice was partially rescued by chronic memantine treatment (Fig. 5a). Dendritic branching plays an important role in propagation of action potentials and synaptic plasticity^39^,^40^, which in turn critically affect learning and memory. In addition, analysis of dendritic spine density of Golgi-stained CA1 pyramidal neurons revealed a significant decrease in db/db mice compared with controls, as reported previously^37^,^38^. This defect in spine density was completely rescued by chronic memantine treatment (Fig. 5b). These results show that chronic memantine treatment of db/db mice increases both dendritic arborization and spine density, suggesting that NMDARs play a crucial role in hyperglycaemia-induced deficits in synaptic structure. Most importantly, these results also indicate that these defects may be therapeutically ameliorated.

### Chronic hyperglycaemia disrupts LTP in an AD mouse model

**Figure 6:**
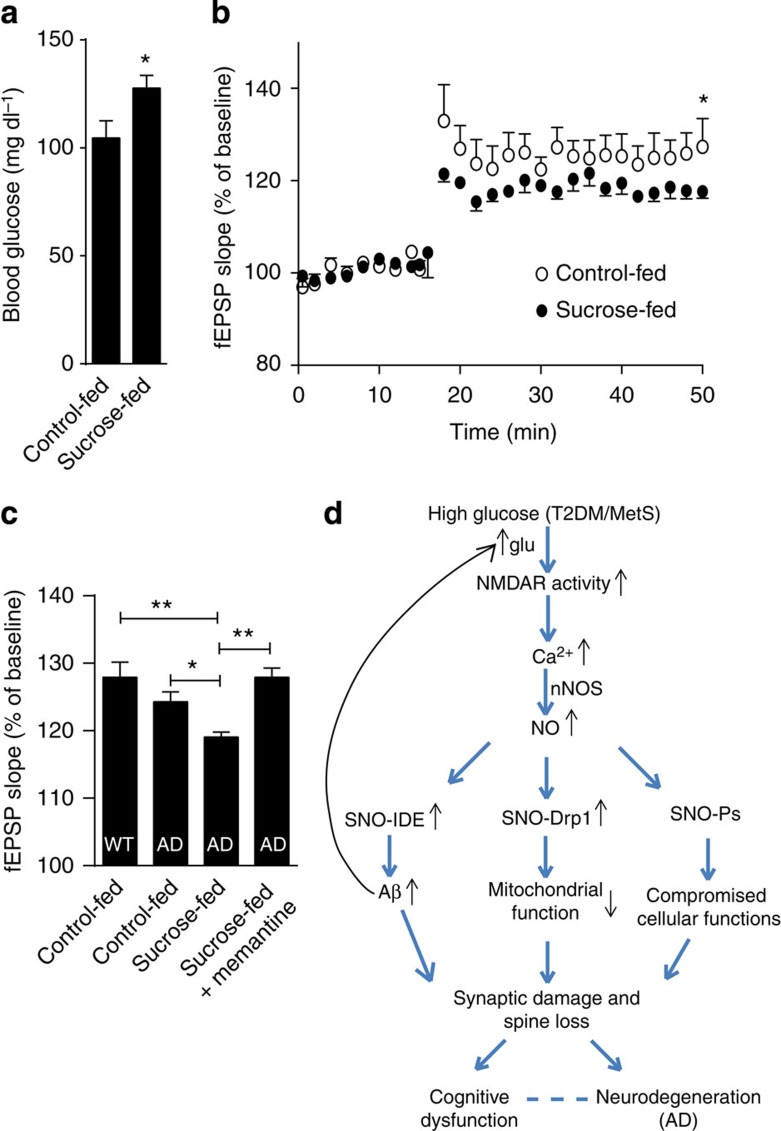
High glucose enhances LTP deficits in the 3 × Tg-AD mouse model. (**a**) Sucrose-fed 3 × Tg AD mice show a significant increase in blood glucose levels compared to controls. Values are mean+s.e.m., *n*=10, *P*<0.05 by Student's *t*-test. (**b**) Hippocampal LTP in 3 × Tg-AD mice. Initial slope of field excitatory postsynaptic potentials (fEPSP) in CA1 pyramidal cells was measured after stimulation of the Schaffer collaterals. Sucrose-fed 3 × Tg-AD mice displayed a significant decrease in LTP compared with controls. (**c**) Percent potentiation of fEPSP over baseline at 50 min after first tetanus in control (WT) and 3 × Tg-AD mice fed with water containing sucrose, sucrose+0.01% memantine (calculated to provide a dose of 2 mg per kg per day), or normal water. Values are mean+s.e.m., *n*≥5 mice per condition; *P<0.05, **P<0.01, by ANOVA with Fisher's PLSD *post hoc* test. (**d**) Schematic of high glucose and Aβ-induced aberrant *S*-nitrosylation mechanisms linking T2DM/MetS to AD. High glucose and oligomeric Aβ lead to increased glutamate (glu) release, stimulation of NMDARs, and increased influx of neuronal Ca^2+^ with consequent NO production. The resulting aberrant *S*-nitrosylation of IDE leads to increased levels of Aβ and insulin, and aberrant *S*-nitrosylation of Drp1 contributes to mitochondrial fragmentation and bioenergetic compromise. These redox-mediated changes, potentially among others (designated SNO-Ps in the figure, indicating other *S*-nitrosylated proteins), culminate in synaptic loss and neurodegeneration, compromising cognitive function in Alzheimer's disease (AD).

Next, to complement our histological findings showing defects in synaptic structure *in vivo*, we studied the functional effects of hyperglycaemia on synaptic plasticity in the setting of AD. Loss of synaptic spines, as a result of nitrosative stress induced by T2DM/MetS and AD, would be expected to decrease synaptic plasticity, thus compromising learning and memory. To monitor the electrophysiological basis for this decrement in synaptic plasticity, we recorded long-term potentiation (LTP) to determine whether it was adversely affected by insults that chronically mimicked features of T2DM/MetS plus AD. For this purpose, we recorded LTP in the CA1 region of cortico-hippocampal slices prepared from the triple transgenic mouse model of AD (3 × Tg-AD)^41^. In these experiments, wild-type or 3 × Tg-AD mice were chronically administered a high-sucrose diet in the drinking water for a 9-month period. Sucrose administration in this manner resulted in chronic hyperglycaemia, similar to that observed in T2DM/MetS (Fig. 6a). We found that the 3 × Tg-AD mice manifested a greater decrement in LTP in the presence of high glucose (Fig. 6b,c). Moreover, memantine prevented the inhibition of LTP, consistent with our findings that this NMDAR antagonist also ameliorated the abnormal increase in [Ca^2+^]_i,_ NO, and synaptic spine loss induced by Aβ and high glucose.

## Discussion

In a number of neurodegenerative conditions manifesting increased RNS, including AD, we and others have shown that aberrant *S*-nitrosylation reactions lead to dysregulated protein activity^10^. Here we found that high glucose, as seen in patient brains with T2DM/MetS, and Aβ oligomers, as observed in human AD brain, coordinately produce a marked increase in RNS and consequent nitrosylation of specific protein thiol targets. For example, glucose and Aβ increase *S*-nitrosylation of IDE, an enzyme known to degrade not only insulin but also Aβ peptide. In neurons, we show that formation of SNO-IDE inhibits degradation of both Aβ and insulin^25^,^26^. This inhibitory effect contributes to increased Aβ levels, thought to be a key contributor to AD^42^,^43^, as well as to increased insulin levels, a known correlate of insulin resistance in T2DM/MetS. This finding is of importance, as brain insulin resistance with compromised insulin signalling was recently demonstrated in human AD patient brains^44^.

In addition, we found that elevated glucose and Aβ result in *S*-nitrosylation of the mitochondrial fission protein Drp1, which contributes to excessive mitochondrial fragmentation, energy compromise, and consequent synaptic dysfunction and loss^30^,^33^. We also show that the increase in [Ca^2+^]_i_ and NO induced by elevated glucose and Aβ that trigger these aberrant nitrosylation events can be attenuated by the NMDAR antagonist memantine in *in vitro* models. Moreover, *in vivo* in the db/db mouse model of T2DM, we demonstrate that memantine can ameliorate dendritic damage and synaptic spine loss.

Importantly, we also found that LTP, a functional index of synaptic plasticity necessary for learning and memory, was most compromised in AD mouse models manifesting concurrent feeding-induced hyperglycaemia. In similar mouse models of AD, sucrose feeding-induced hyperglycaemia has been shown to exacerbate deficits in spatial learning and memory^12^,^45^. In our experiments, the decrease in LTP was rescued by treatment with memantine. In a correlative manner, human phase 3 clinical trials of memantine have been shown to transiently stabilize or improve behavioural deficits in AD^46^. Our findings thus provide mechanistic insight into the molecular and electrophysiological events underlying the neurobehavioral deficits in hippocampal-dependent learning and memory induced by hyperglycaemia in the setting of AD.

Collectively, our results suggest that increased levels of glucose, as seen in T2DM/MetS, and oligomeric Aβ peptide, as observed in AD, compromise neuronal form and function via oxidative pathways in molecular metabolism. While other aberrant nitrosylation pathways are also likely to occur under these conditions, we identify two nitrosylated proteins, Drp1 and IDE, which can directly contribute to disease pathogenesis and progression. In addition, ROS (for example, O_2_^−^) and RNS (for example, NO) can react to form peroxynitrite, resulting in nitrotyrosinated proteins, and this mechanism may also potentially contribute to redox-mediated dysfunction in this setting^47^. In summary, these findings delineate a redox-mediated pathway whereby high glucose and oligomeric Aβ coordinately induce nitrosative stress in an NMDAR-dependent manner that ultimately results in synaptic defects, as shown in both T2DM and AD mouse models (Fig. 6d). The results have both mechanistic and therapeutic implications for T2DM/MetS patients with cognitive decline because memantine, an uncompetitive NMDAR antagonist that is already approved by the Food and Drug Administration for treatment of moderate-to-severe AD, attenuates high glucose/Aβ-induced nitrosative stress that contributes to defects in synaptic function.

## Methods

### High glucose concentrations and oligomeric Aβ preparations

In each model system, ‘high glucose' was engendered by raising the glucose 15–25 mM over ambient conditions. This level was chosen after pilot studies determined a far-ranging dose response to increasing levels of glucose and since it represents blood glucose of ∼270–450 mg dl^−1^, simulating moderate-to-severe T2DM/MetS. To control for potential osmotic effects of this increase in glucose, we used equimolar mannitol. Human recombinant Aβ_1-42_ oligomers were prepared, characterized, and quantified as we have recently described in detail^9^, in Supplementary Information). We chose an exposure of 250 nM recombinant oligomeric Aβ_1–42_ based on our prior studies with both recombinant and naturally occurring Aβ peptides^9^.

### Cell culture and transfection

Primary cortical neurons were isolated from E16-18 Sprague-Dawley rats and maintained in culture as described previously^20^. In brief, dissected cerebrocortices were treated with trypsin, and dissociated cells were plated on poly-L-lysine coated plates in D10C medium containing 25 mM glucose. Experiments were performed 14–21 days after plating. Primary cortical cultures were transfected using Lipofectamine 2000 (Invitrogen). SH-SY5Y cells were maintained in DMEM (Sigma) containing 10% fetal bovine serum, 50 IU ml^−1^ penicillin (Omega Scientific) and 50 μg ml^−1^ streptomycin (Omega Scientific) in a humidified atmosphere of 5% CO_2_. Cells were transfected using Lipofectamine LTX plus (Invitrogen) according to the manufacturer's instructions.

### Generation of hiPSCs and neuronal differentiation

To generate hiPSCs from human dermal fibroblasts (ATCC, Catalogue no. CRL1634) we used an integration-free reprogramming method, transfecting fibroblasts with episomal expression vectors collectively encoding six reprogramming factors: OCT3/4, SOX2, KLF4, L-MYC, LYN28, and p53-shRNA^48^. hiPSC colonies were maintained on mouse embryonic fibroblast feeders and were validated for pluripotency, trilineage differentiation capability, and karyotypic stability as we have previously described^49^. Neuronal differentiation was performed using a protocol modified from Chambers *et al*^50^. Briefly, feeder-free hiPSCs were treated with small-molecule inhibitors of bone morphogenetic protein (Dorsomorphin), Activin/Nodal (A83-01), and Wnt/β-catenin (PNU-74654) for 1 week. The resulting PAX6+ cells were cultured for 2–3 weeks as floating neurospheres in DMEM/F12 medium supplemented with N2 and B27 (Invitrogen), in the presence of basic FGF (20 ng ml^−1^). Cells subsequently adhered to form a monolayer on *p*-ornithine/laminin-coated dishes, and the resulting neural rosettes were manually isolated and expanded. Cells were then seeded onto glass coverslips (7 × 10^5^ cells per cm^2^) for terminal differentiation into mature neurons in the presence of medium supplemented with N_2_, brain derived neurotrophic factor (10 ng ml^−1^), glial cell line-derived neurotrophic factor (10 ng ml^−1^), and dibutyryl-cAMP. Neurons were cultured on a bed of astrocytes (1:3 astrocytes to neurons) for the experiments described here.

### Cortico-hippocampal slice preparation

C57Bl/6 male mice at 2–3 months-of-age were anaesthetized using isoflurane and decapitated as per Institutional approval by the Animal Care Committee. The brain was rapidly dissected and cortico-hippocampal slices (350 μm) were collected in ice-cold dissection buffer as previously described^51^. Slices were then placed at 30 °C for 1 h for recovery in ACSF containing (in mM): 124 NaCl, 5 KCl, 26 NaHCO_3_, 1.25 NaH_2_PO_4_, 2 CaCl_2_, 1 MgCl_2_, and 10 glucose. ACSF and dissection buffer were bubbled with 95% O_2_/5% CO_2_.

### Immunoblot analysis

For immunoblot analyses, samples were separated on 4–12% gradient NuPAGE gels (Invitrogen) and transferred to nitrocellulose membranes. Primary antibodies for immunoblots included anti-IDE antibody (1:1,000 dilution, Covance) and anti-Drp1 antibody (1:1,000 dilution, BD Transduction Laboratories). Secondary antibodies were purchased from Amersham Biosciences. Labelled proteins were detected using the ECL-plus detection system (Amersham Bioscience) or SuperSignal West Dura Extended Duration Substrate (Pierce). Immunoblot images of Figs 2a,c–e and 3b,c, and Supplementary Fig. 1 were cropped for presentation. Full-immunoblot images are provided in Supplementary Fig. 2.

### DAF-FM imaging for nitric oxide

DAF-FM diacetate, a cell-permeable indicator^52^,^53^ was used to monitor NO in acute cortico-hippocampal slices and cultured primary cortical cultures. Slices were prepared in dissection buffer and transferred to ACSF containing either 10 or 25 mM glucose in the presence or absence of 250 nM oligomeric Aβ. Dissection buffer was the same as described above. After 1 h at 30 °C, slices were incubated with ACSF containing 2.5 μM DAF-FM diacetate for 15 min at room temperature in the dark, which was then washed twice with ACSF. Slices were then imaged under epifluorescence deconvolution microscopy. Image analysis was performed using SlideBook software (Intelligent Imaging Innovations) to obtain values of fluorescence intensity.

Cortical cultures plated on coverslips were incubated with 5 μM DAF-FM diacetate and 1 mM L-Arg in buffer based on prior composition^9^ for 30 min at room temperature in the dark. Cultures were then washed twice with buffer, imaged in time-lapse mode using a CCD camera mounted on a Zeiss Axiovert 35 microscope, and analysed with SlideBook software. Values of fluorescence intensity change were calculated as Δ*F*/*F*_0_ and plotted as a fraction of 100.

### [Ca^2+^]_i_ measurements

Primary rat cortical cells (14–21 days in culture) were loaded for 2 h at room temperature with fura-2/AM (4 μM) in imaging buffer^9^. After loading, the cells were washed twice with imaging buffer. For fura-2-based measurements of [Ca^2+^]_i_, we used a liquid light guide connected to an ultrahigh-speed wavelength switcher fitted with a 175 Watt xenon arc lamp (Lambda DG4; Sutter Instrument) and an optimized fura-2 filter set for obtaining the 350/380 nm excitation ratio. Images were collected using a CCD camera mounted on a Zeiss Axiovert 35 microscope and analysed with SlideBook software. Fluorescence values were calculated as changes in fluorescence ratios relative to baseline fluorescence intensity (Δ*F*/*F*_0_). For each experiment, values were calculated by dividing the change in peak fluorescence (Δ*F*) by the resting baseline fluorescence (*F*_0_); thus, fluorescence values represent fractional changes above baseline (Δ*F*/*F*_0_). These values were plotted as a.u.

### Biotin switch assay

Analysis of SNO-IDE and SNO-Drp1 by the biotin switch assay was performed as previously described^20^,^27^. After cell lysis, free thiols were blocked with methyl-methanethiosulfonate. Cell extracts were precipitated with acetone and resuspended in HEN buffer with 1% SDS. Nitrosothiols were selectively reduced by ascorbate to reform the thiol group and subsequently biotinylated with 1 mM biotin-HPDP (Pierce, Rockford, IL). Controls were performed without ascorbate to ensure specificity of the observed biotinylated bands. The biotinylated proteins were pulled down with streptavidin–agarose beads and analysed by immunoblotting.

### FAβB-degradation assay

Cortical neurons or SHSY-5Y cells exposed to old or freshly prepared SNOC were lysed in PBS containing 0.01% Triton X-100 and protease inhibitor cocktail. Cell lysates containing 100 μg of protein were mixed with 3 μM fluorescein-Aβ-Biotin or 5-carboxyfluorescein (FAM)-Aβ-Biotin (FAβB) in the presence or absence of IDE inhibitor. Reaction mixtures (100 μl) were incubated at 37 °C for 20 min and quenched by addition of phosphate buffered saline (700 μl) containing 1, 10-Phenanthroline (2 mM). Uncleaved FAβB substrate was precipitated from each quenched reaction by addition of Neutravidin-coated agarose beads (Pierce) followed by gentle rocking for 30 min and centrifugation at 14,000 × *g* for 10 min. Supernatant (containing cleaved fluorescein-Aβ fragments) was carefully transferred as three 200- μl aliquots into black 96-well plates (Nunc), and fluorescence intensity (488 excitation/515 emission for Fluorescein and 494 excitation/521 emission for FAM) was measured with a fluorescence plate reader.

### Human brain samples

All the post-mortem brain samples were a kind gift from Eliezer Masliah in the Neuropathology Core at the University of California, San Diego. These samples were obtained and analysed with Institutional permission following State of California and NIH guidelines. Informed consent was obtained according to procedures approved by the Institutional Review Board at the University of California, San Diego School of Medicine. All samples were obtained from the frontal cerebrocortex of post-mortem specimens of patients diagnosed with AD or age-matched non-CNS disease control subjects. Patient data are summarized in Supplementary Table 1.

### Analysis of dendritic spine density in organotypic slices

Thy1-YFP transgenic mice (8–10 days old) were anaesthetized by placing on ice and decapitated, as per Institutional approval by the Animal Care Committee. The brain was rapidly dissected out, and cortico-hippocampal slices (350-μm thick) were collected in ice-cold dissection buffer bubbled with 95% O_2_/5% CO_2_. Slices were transferred onto a Millicell cell culture insert (Millipore), and submerged in standard media^9^. Thereafter, 50% of the culture medium was replaced every 2–3 days with fresh medium. Slices were exposed to oligomerized Aβ_1–42_ peptide, high glucose in the presence and absence of 1 mM L-NAME for 7 days, which were replenished with each medium change.

Evaluation of dendritic spine density was performed as described previously^9^,^54^. Briefly, slices were fixed in 4% paraformaldehyde, and images of YFP-labelled dendritic spines were acquired by deconvolution epifluorescence microscopy using SlideBook software. For YFP-expressing neurons (*n*>12 for each condition), two distinct fields of secondary or tertiary dendrites, at least 40 μm in length, were randomly selected and analysed in a masked manner using the SlideBook analysis programs.

### Measurement of oxygen consumption rate

Oxygen consumption rate was analysed in an XF24-3 Extracellular Flux Analyzer (Seahorse Biosciences). After 11 days in culture, rat cortical neurons were exposed to high glucose (or osmotic control), Aβ, or both for 3 days at 37 °C. For assessment of oxygen consumption rate, three baseline recordings were made, followed by sequential injection of the ATP synthase inhibitor oligomycin and the mitochondrial uncoupler *p*-triflouromethoxyphenylhydrazone to monitor maximal respiratory capacity. Data were normalized to protein concentration in each well.

### Memantine treatment and dendrite and spine analysis *in vivo*

All animal experimental procedures were reviewed and approved by the Institutional Animal Care and Use Committee at the Sanford Burnham Prebys Medical Discovery Institute. One-month-old T2DM (db/db) male mice were treated with memantine (1 mg kg^−1^, intraperitoneal) twice daily for 3 months. Mice were then anaesthetized using avertine and transcardially perfused using saline solution. Following saline perfusion, mouse brains were collected and transferred to Golgi impregnation solution for Golgi-Cox impregnation using the FD Rapid GolgiStain kit (FD NeuroTechnologies, Inc.) according to the manufacturer's protocol. Following impregnation, 150 μm thick brain slices were cut using a vibratome, which were then stained according to the manufacturer's protocol. All subsequent analyses were performed by individuals without knowledge of the treatment group.

For Sholl analysis, Golgi-stained slices were imaged to generate three-dimensional (3D) montages of entire CA1 hippocampal neurons at 20 × resolution under deconvolution microscopy using SlideBook 5.0 software (Intelligent Imaging Innovations). Neurolucida neuron tracing software (MBF Bioscience) was used to delineate the whole-cell profile, and Sholl analysis was performed using Neurolucida Explorer analysis software. Dendritic maxima (representing the maximum number of dendritic crossings) and critical values (radius at which dendritic maxima occur) of each cohort were compared.

For dendritic spine analysis, Golgi-stained slices were imaged to generate 3D montages of secondary or tertiary dendrites of CA1 hippocampal neurons at × 63 resolution under deconvolution microscopy using SlideBook 5.0 software. Dendrites at least 30 μm in length were randomly selected and analysed using SlideBook analysis programs.

### AD transgenic mouse model

Triple transgenic AD male mice^55^ were bred for at least eight generations on the C57/BL6 background and randomized to three treatment groups. Starting at 3 months of age and continuing until 12 months of age, Group one drank water containing 2% sucrose; Group two, 0.01% memantine dissolved in 2% sucrose; and Group three, standard water. Monitoring water bottle volume revealed that there was no significant difference in water intake among the various groups. Two days before killing, all animals were given standard water to allow memantine to clear their systems before the electrophysiological experiments and thus to observe lasting phenotypes compared with age- and sex-matched control mice. Blood glucose level and weight were recorded before killing.

### Multielectrode array electrophysiological recordings of LTP

Mice underwent cervical dislocation before decapitation following National Institute of Health and Institutional guidelines. Hippocampi were dissected in standard ACSF medium^51^. Hippocampal slices (300 μm in thickness) were sectioned transversely with respect to the longitudinal axis using a vibratome (VT1000S, Leica, Germany) and incubated at room temperature in ACSF aerated with 95% O_2_ and 5% CO_2_ (ref. 51). Slices were equilibrated for ≤1 h on the multielectrode array chamber (MEA60 Multi Channel Systems, Reutlingen, Germany) prior to recording. Slices were held in place with a nylon mesh and perfused with ACSF at 32 °C for the duration of the experiment. Field excitatory postsynaptic potentials were evoked in CA1 pyramidal neurons by monopolar biphasic voltage stimulation delivered at the Schaffer collaterals. Increasing voltage stimulation was used to determine the threshold at which 30% maximal response was obtained in the absence of a population spike. Baseline recordings were obtained with 30 stimuli at 30 s intervals. Potentiation was induced by four bursts at 20-s intervals with each burst consisting of ten stimulatory pulses at 100 Hz. Field excitatory postsynaptic potentials amplitude and initial slope were analysed at the apical dendrites (stratum radiatum). Per cent potentiation was calculated as the ratio of the mean field excitatory postsynaptic potentials initial slope, recorded 50 min after first tetanus stimulation, and divided by the mean field excitatory postsynaptic potentials initial slope of the baseline recording.

### Statistical analysis

Data are presented as mean±s.e.m. Statistical analyses were performed using Graphpad Prism software. Statistical significance was determined by Student's *t*-test for single comparisons or analysis of variance (ANOVA) for multiple comparisons with appropriate *post hoc* testing. Sample sizes for all experiments were determined by power analyses based on previously published data from our laboratory; previous data also were used to determine whether a one-way or two-way statistical test was employed.

## Additional information

**How to cite this article:** Akhtar, M. W. *et al.* Elevated glucose and oligomeric β-amyloid disrupt synapses via a common pathway of aberrant protein *S*-nitrosylation. *Nat. Commun.* 7:10242 doi: 10.1038/ncomms10242 (2016).

## Supplementary Material

Supplementary Information Supplementary Figures 1-2 and Supplementary Table 1.
