# Supplementary material for: Elevated glucose and oligomeric β-amyloid disrupt synapses via a common pathway of aberrant protein S-nitrosylation
Source: Nat Commun. 2016 Jan 8;7:10242. doi: 10.1038/ncomms10242 (PMC4729876; doi:10.1038/ncomms10242)
Supplement: Supplementary Information — Supplementary Figures 1-2 and Supplementary Table 1. [file ncomms10242-s1.pdf]

Supplementary Figure-1 Lipton

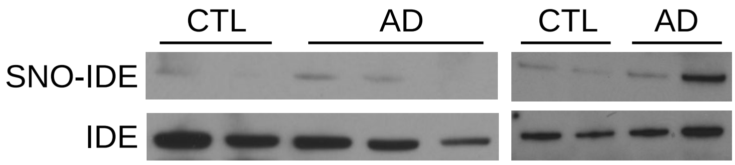

Supplementary Figure 1: Detection of S-nitrosylated (SNO)IDE in human postmortem brain lysates by biotin-switch assay. CTL = Control patient lysates AD = Alzheimer's disease patient lysates

Supplementary Figure-2 Lipton

Figure 2A

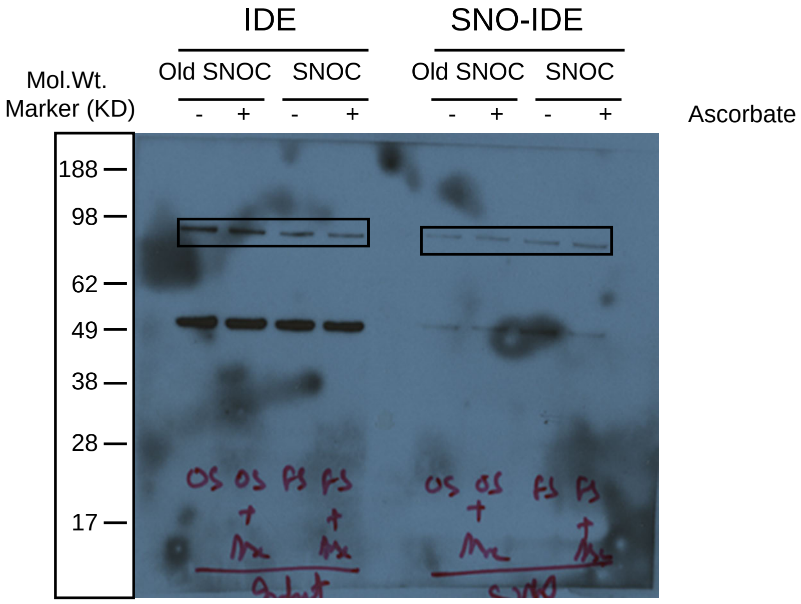

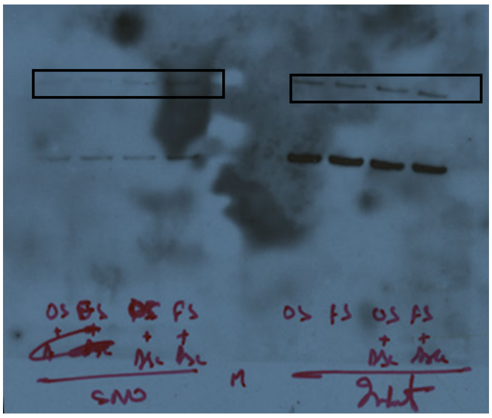

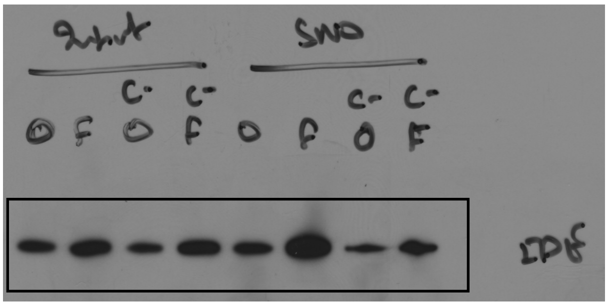

Supplementary Figure 2: Original Scaned Western Blots

Figure 2C

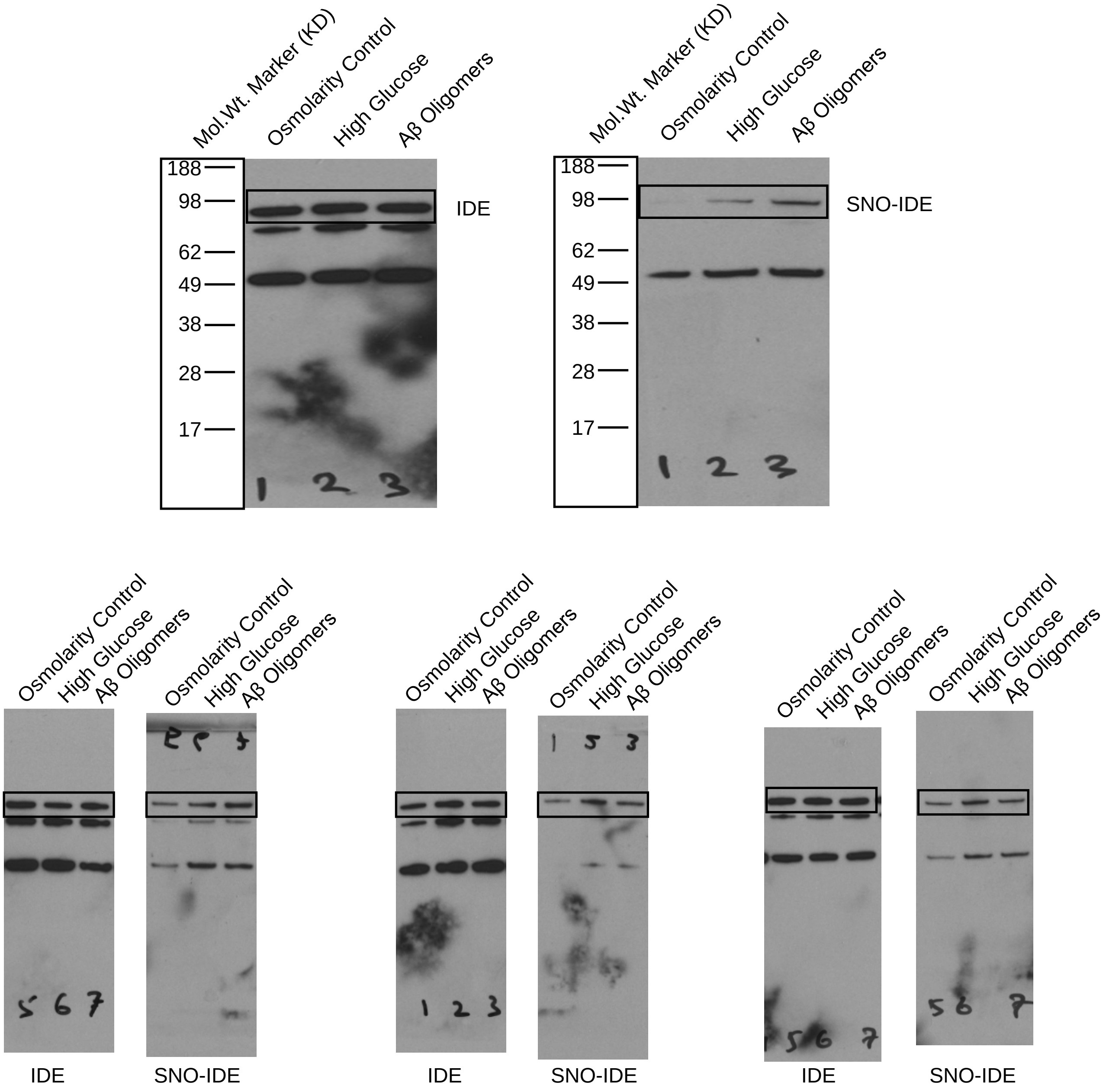

Rat Cortical Cultures

Supplementary Figure 2: Continued

Figure 2C

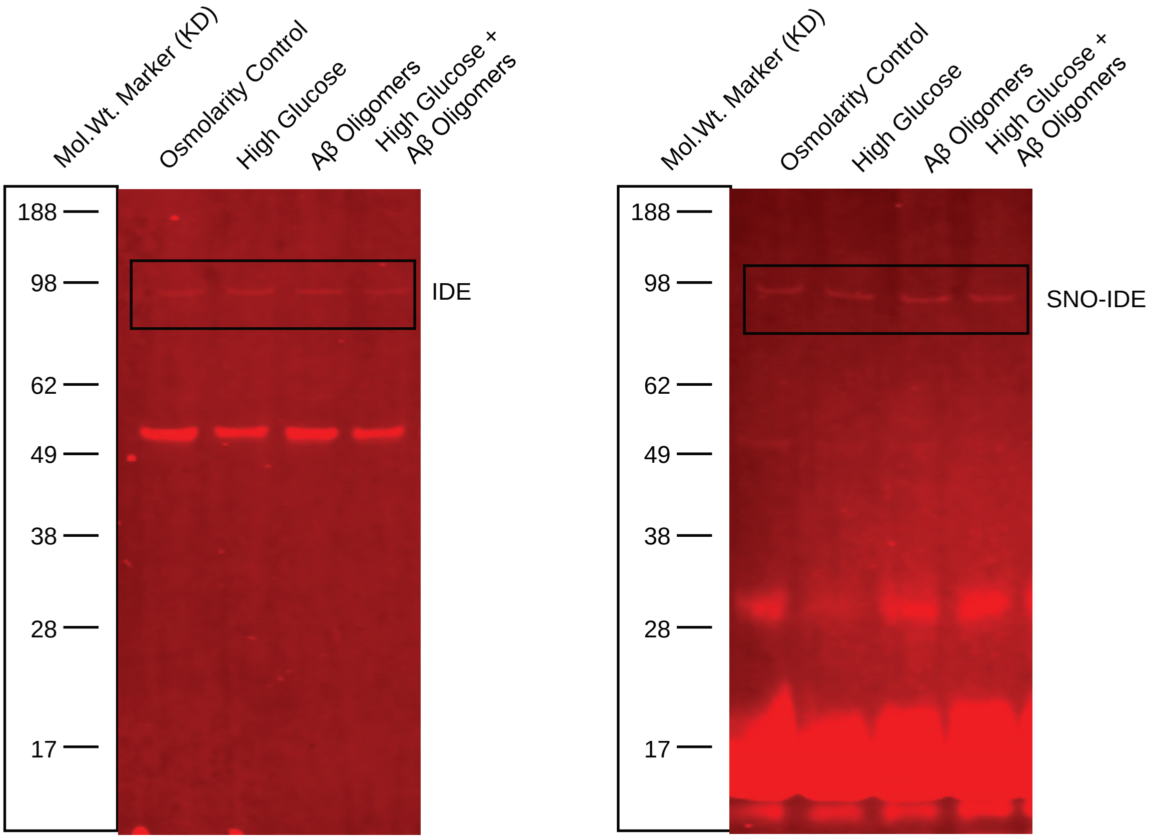

hiPSC-derived Cortical Cultures

Supplementary Figure 2: Continued

Figure 2D

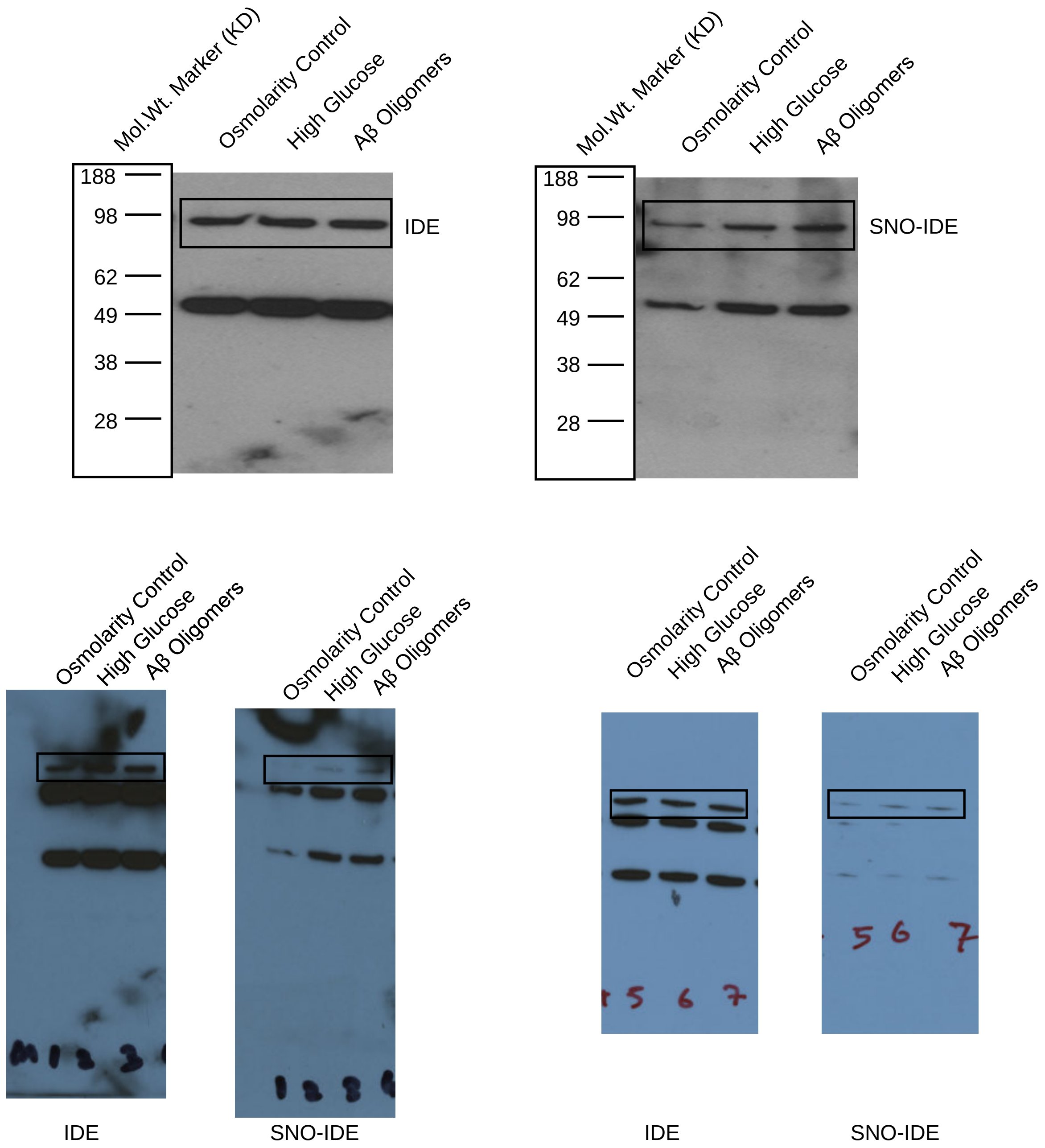

Supplementary Figure 2: Continued

Figure 2E

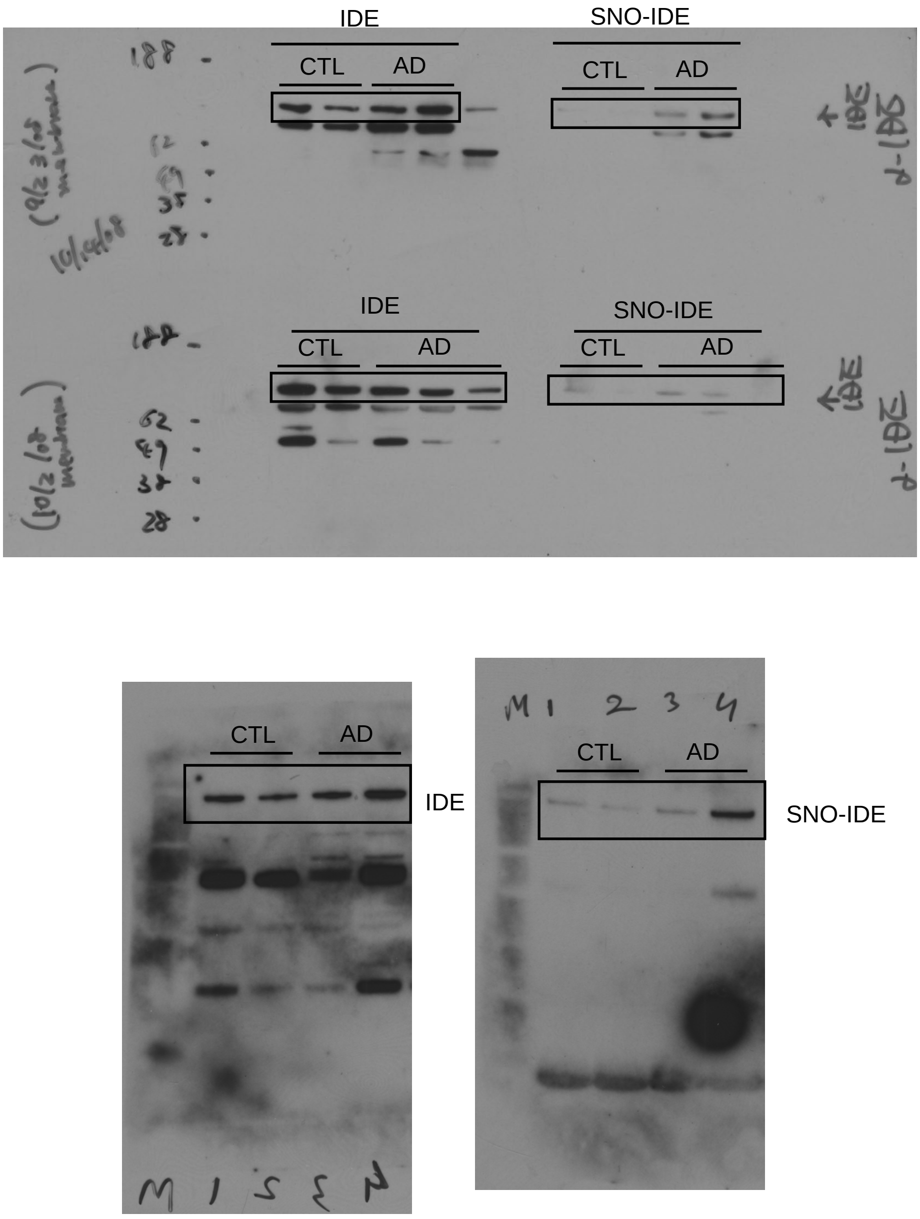

Supplementary Figure 2: Continued

Figure 3B

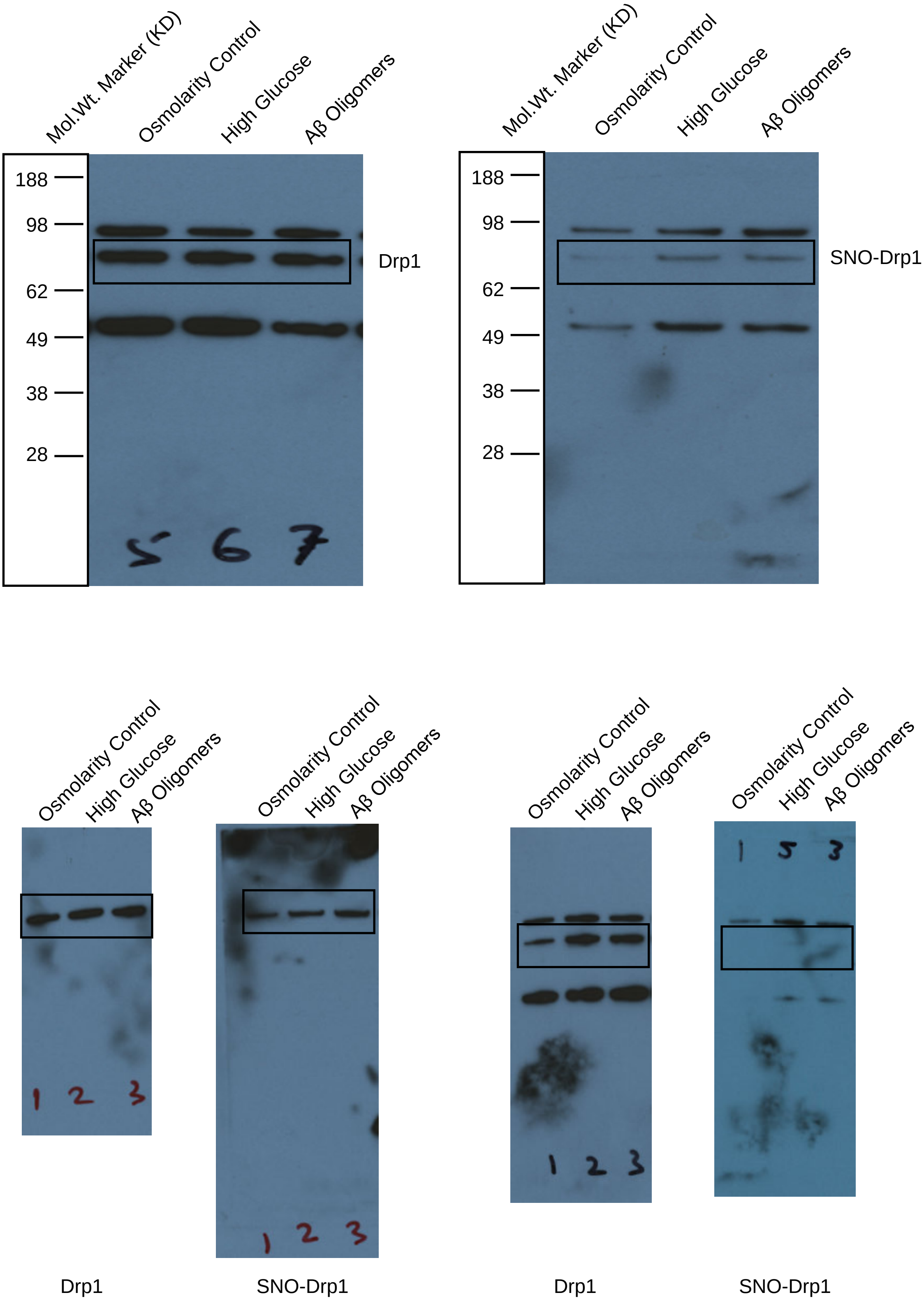

Supplementary Figure 2: Continued

Figure 3C

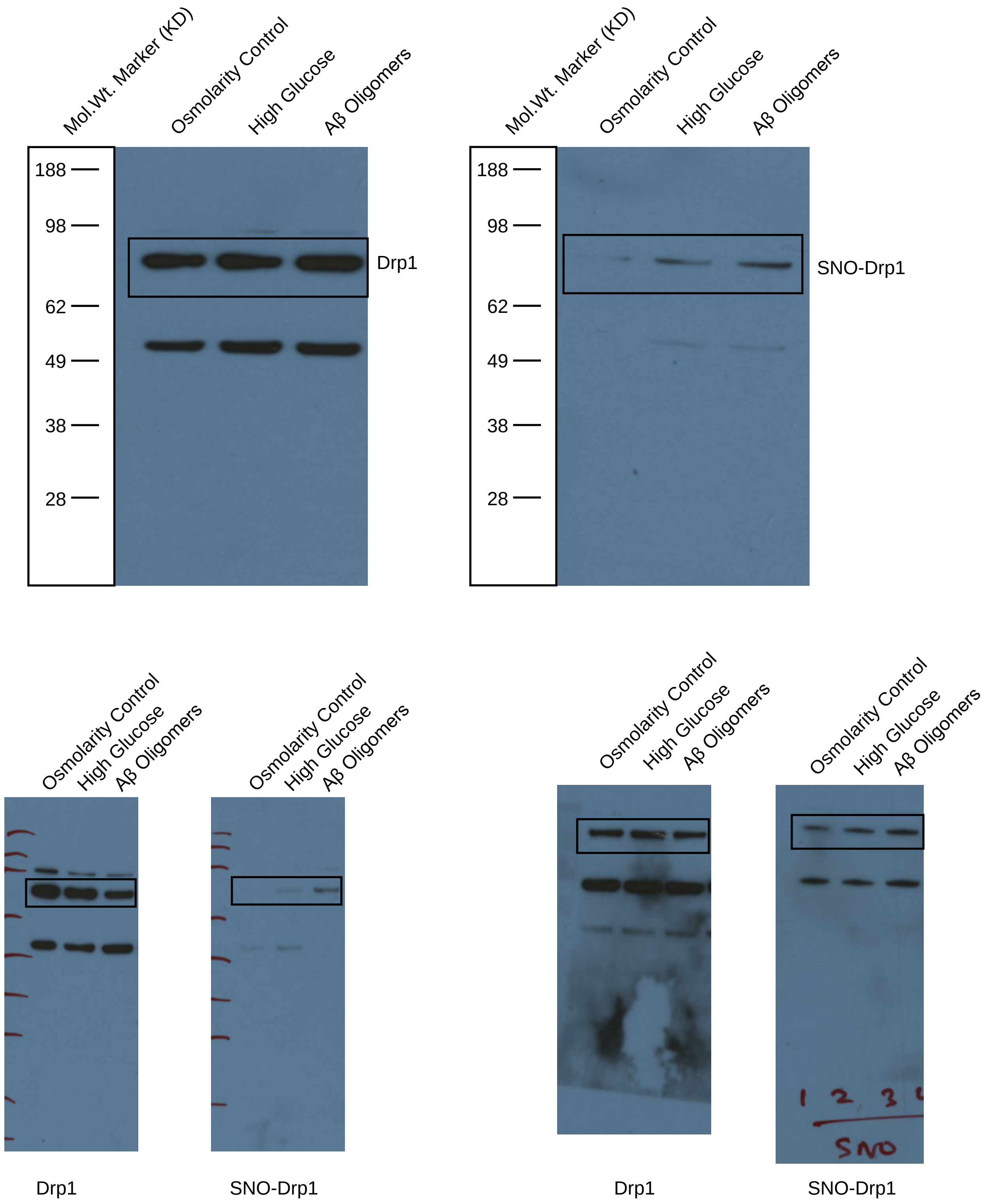

Supplementary Figure 2: Continued

	Age	Sex	PMI (Hours)
**Control**	71	Male	2
**Control**	102	Female	9
**Control**	71	Male	2
**Control**	91	Male	6
**Control**	71	Male	2
**Control**	87	Male	N/A
**AD**	80	Female	N/A
**AD**	41	Female	10
**AD**	65	Male	N/A
**AD**	77	Male	4
**AD**	83	Female	N/A
**AD**	79	Male	4
**AD**	91	Male	N/A

Supplementary Table 1. Postmortem human brain samples used in this study. Control patients died of non-CNS causes and did not have Type 2 diabetes mellitus/Metabolic syndrome (T2DM/MetS) or Alzheimer's disease (AD) by clinical or pathological criteria. AD cases met the criteria for MetS or had treated T2DM in addition to AD. PMI, Post-Mortem Interval; AD, Alzheimer's disease samples.
